# Association between Drug Usage and Constipation in the Elderly Population of Greater Western Sydney Australia

**DOI:** 10.3390/ijerph15020226

**Published:** 2018-01-29

**Authors:** Alexandra Fragakis, Jerry Zhou, Haider Mannan, Vincent Ho

**Affiliations:** 1 School of Medicine, Western Sydney University, Campbelltown, Sydney, NSW 2560, Australia; 17513311@student.westernsydney.edu.au (A.F.); V.ho@westernsydney.edu.au (V.H.); 2 Translational Health Research Institute, Western Sydney University, Campbelltown, Sydney, NSW 2560, Australia; h.mannan@westernsydney.edu.au

**Keywords:** constipation, prescription drug, elderly, population study

## Abstract

The low socioeconomic region of Greater Western Sydney (GWS) has higher than average rates of gastrointestinal symptoms. The relationship between prescription drug usage and constipation has not been explored. The aim of this study was to investigate the impact of drug use on constipation in the elderly population of GWS (NSW, Australia). A random selection of elderly residents completed a postal questionnaire for constipation and drug use (response 30.7%). Bivariate associations between constipation and number of drug use and number of drug use with constipation adverse effect were compared. For multivariate analysis multiple logistic regression was performed for constipation with the number of drugs, use of drugs with known constipation side effects, and each drug class (Anatomical Therapeutic Chemical Classification System (ATC) level 4) as independent variables. The prevalence of constipation was 33.9%. There was a dose–response relationship between constipation and the number of drugs used (odds ratio 1.24, *p* < 0.001) and the usage of drugs with known constipation adverse effects (odds ratio 2.21, *p* = 0.009). These findings suggest that constipation is associated with the number of drugs used, particularly those with constipation adverse-effects, in the elderly of GWS.

## 1. Introduction

Constipation is a common problem worldwide. Constipation has a significant impact on quality of life but only a minority of patients suffering from constipation seeks health care. The elderly are more susceptible to constipation, with an estimated prevalence of chronic constipation globally at 30% in community-dwelling adults aged over 65 [1,2,3]. Although many constipation risk factors are well known, the role drugs and polypharmacy has on constipation in elderly remains controversial. Several studies have identified polypharmacy as a risk factor for increased drug adverse effects [4,5] including English and American studies that specifically identified polypharmacy as a risk factor for constipation [6,7]. On the other hand, studies within the Norwegian [8,9] and Dutch [10] populations did not find constipation to be associated with polypharmacy, but rather a small specific group of drugs with constipation adverse effects. What is known is that the use of prescription drugs in elderly has more than doubled from 1992 to 2006 [11,12] and polypharmacy is fast becoming a global issue.

In countries with social health programs, those with low socioeconomic status are more likely to receive a greater number of drugs, more generic products and be exposed to major polypharmacy compared with the affluent patients [13,14,15,16,17]. While polypharmacy may be a necessity in the management of some medical conditions, there is an increased risk of adverse drug events [5] with constipation being one of the most common [18]. Within these communities, the elderly are most likely to be dependent on multiple drugs and are more susceptible to adverse drug constipation due to compromised physiological regulation of gastrointestinal transit and associated anorectal motor functions [19,20,21].

Greater Western Sydney (GWS) is home to half the population of Sydney and is classified as a low socioeconomic region. The low socioeconomic status of GWS has predisposed its elderly population to poorer health outcomes [13,14]. The prevalence of constipation in the demographic of GWS (10.3–30.1%) has been found to be higher than the rest of Sydney (6.3–20.6%) [22,23]. Constipation as an adverse effect of medication usage has neither been studied in GWS nor in Australia.

This cross-sectional study aims at determining the associations between drug usage and constipation in the community-dwelling elderly population of GWS (NSW, Australia).

## 2. Materials and Methods

### 2.1. Study Population

From 2015 to 2016, this cross-sectional study was performed in community dwelling residents over 65 in GWS region, NSW, Australia. GWS was defined in accordance with the Australian Bureau of Statistics as the areas defined by the Western Sydney Regional Organisation of Councils Region, the Macarthur Regional Organisation of Councils Region and The Hills Shire. The Australian Electoral Commission provided the names and residential addresses of a sample of 1100 randomly selected electors, with an equal number of men and women, who were aged over the age of 65 as of 1 January 2016. A questionnaire requesting general health information, Rome III criteria for constipation [24], and drugs taken was sent to all subjects. A return addressed envelope was included for participants to respond. This study was approved by the Western Sydney University Human Research Ethics Committee, approval number H11369.

### 2.2. Variables

Age and gender were recorded. Constipation was defined according to Rome III criteria for functional constipation except for insufficient criteria for irritable bowel syndrome because cognitive impairment made this information unreliable, and/or regular use of a laxative. Drugs were measured as use of drugs and the number of drugs (laxatives Anatomical Therapeutic Chemical Classification System (ATC)-class A06, dermatologicals, and topical preparations for eyes and ears were excluded). The drugs were grouped according to the ATC at level 4 since most drugs at this level have common adverse drug reactions [25]. Drug classes known to cause constipation, as described by O’Mahoney et al. [26], were recorded as “use of one or more drugs with known constipation adverse-effects”.

### 2.3. Statistical Analysis

Bivariate associations between constipation and the total number of drugs used or number of drugs with known constipation side effects were analyzed with chi-squared test for trend while those between constipation and age was analyzed using the nonparametric Mann–Whitney *U* test depending on the non-normal distribution of age. Bivariate associations between constipation and the use of any drugs, use of drugs with constipation adverse-effect, regular use of laxatives, drugs with constipation adverse effects and age were analyzed with either Fisher’s exact test or chi-squared test depending on data sparsity. For quantitative variables which are skewed we calculated semi-interquartile range as a measure of variability while for quantitative variables which are symmetrical we calculated standard deviation as a measure of variability. Multivariable analysis using logistic regression was performed with constipation as dependent factor and either number of drugs, drugs with known constipation side-effects, or groups of drugs at ATC-level 4 with *p* ≤ 0.2 in bivariate analysis as independent variables. Both age and gender were included in the multivariate analysis. Two-sided *p* values ≤ 0.05 were regarded as statistically significant and *p*-values ≤ 0.10 as a trend [9]. SPSS Statistics 24 (SPSS Inc., Chicago, IL, USA) was used for the analysis.

## 3. Results

Out of the 1110 surveys mailed out to elderly residents of GWS, 385 (34.7%) were returned however, 149 surveys were excluded from the analysis as either the candidate did not meet the selection criteria or did not complete the survey. A total of 236 valid surveys were used for the analysis (21.3%). Table 1 and Table 2 show the characteristics of the residents and their use of medications. The prevalence of constipation was 33.9%. The proportion of the GWS elderly population that used one or more drugs was 88.9% and the median number of medications was 4.0 (range 0–16); while 112 (47.4%) participants used one or more drugs with known constipation side-effects.

Table 1 and Table 2 give comparisons between residents with and without constipation. The numbers of drugs as well as the usage of drugs with constipation adverse effects were statistically significantly associated with constipation. Opioids (N02A), drugs for peptic ulcer and gastroesophageal reflux disease (GERD) (A02B), antithrombotic agents (B01A), other analgesics and antipyretics (N02B) were significantly associated with constipation.

Table 3 gives the independent predictors for constipation using logistic regression analysis. A strong associated was observed between constipation and use of drugs with constipation adverse effect odds ratio (OR) 2.21; 95% confidence interval (CI) 1.20–4.00. The OR for numbers of total drugs and number of drugs with adverse-effects were 1.24 (95% CI 1.13–1.35) and 1.68 (95% CI 1.25–2.26), respectively. Calcium supplements (A12A) and angiotensin converting enzyme (ACE) inhibitor (C09A) were not independent risk factors for constipation at 5% level of significance. While drugs for peptic ulcer and GERD (A02B), antithrombotic agents (B01A), other analgesics and antipyretics (N02B) were independent protective factors for constipation having strong effect sizes 0.23, 0.33 and 0.35, respectively, indicating 77%, 67% and 65% reductions in odds of constipation.

## 4. Discussion

This study found that constipation was more prevalent in the elderly population of GWS (33.9%) than in similar demographic groups across Sydney (30.7%) [22] and nationally in Australia and New Zealand (20.3% and 27.7%, respectively) [3,27]. Corresponding international studies on the prevalence of constipation within community dwelling elderly include the following prevalence: 12% Singapore [28]; 23% Scotland [29]; 12.7%, 22.6% and 39.5% United States [1,7,30]; and 38% Finland [31]. The prevalence of constipation in our study was remarkably similar to studies in North America and Northern Europe, while prevalence of constipation was much lower in Southeast Asia. Low socioeconomic status plays a significant role in increasing the prevalence of constipation in all global regions [32]. Within Sydney, study by Bytzer et al. [23] found adults in the Penrith and Blue Mountains, regions of GWS, had an overall higher reporting of constipation than the national average. They postulated that risk factors for constipation, such as poor diet and obesity, physical inactivity, the use of medications and smoking, are likely to be unevenly distributed across social classes and this may explain the higher prevalence of constipation in the low socioeconomic class. Our findings confirm that medication usage and constipation rates were both higher in GWS than the national average, with the usage of drugs demonstrated to be a risk factor towards constipation.

Comparison of constipation rates can be difficult due to variations in the definitions of constipation. This study uses a combination of regular use of laxatives and Rome III criteria for functional constipation and constipation-predominant irritable bowel syndrome (IBS). IBS is a wide spectrum gastrointestinal disorder characterized by abdominal discomfort and altered bowel function; including constipation predominant symptoms. Patients using laxatives regularly are categorized to have constipation although they do not fulfil the Rome III criteria, and the two Rome III groups (functional constipation and constipation-predominant IBS) are difficult to distinguish. The issue of overlap between constipation-predominant IBS and functional constipation has been examined in detail recently [33]. The authors suspended the mutual exclusivity of the two sets of diagnostic criteria, and reported that this led to significant overlap between them, implying that constipation-predominant IBS and functional constipation may be different subgroups within the same disorder. The validity of the Rome III criteria for constipation has been questioned, but no other instrument is available [34]. Our definition is therefore sensible in this study population.

### Drugs and Constipation

The main finding was that the number of drugs and usage of drugs with constipation adverse-effect were significantly associated with constipation in the elderly population of GWS. The percentage of elderly on at least one drug and average number of drugs in our study (88.9%, four drugs per person) was higher than that of elderly nationwide around Australia (85%, 3.8 drugs per person) [35] but lower than Sydney nursing home residents (97% on at least one drug, five drugs per resident) [36].

Our results showed that while the use of drugs was not associated with constipation, the number of different drugs was a relative risk factor 1.24 (95% CI 1.13–1.35). Given the worldwide rise of persons over the age of 65, polypharmacy is becoming more prevalent in older adults [37,38]. Current medical practice guidelines often require multiple medications to treat each chronic disease state for optimal clinical benefit. Therefore, an elderly patient with at least two disease states will usually exceed the threshold for polypharmacy of five or more drugs [39]. Cross-sectional studies of community dwelling for the elderly in the United States report 36.6% took at least five prescription medications in 2006 [12], compared to 14 years ago in 1992 where only 10.5% of the same population were classified as polypharmacy [11].

A population-based study of outpatient clinic and emergency department visits found outpatients taking five or more drugs had an 88% increased risk of experiencing an adverse drug effect compared to those who were taking fewer drugs [40]. The increased use of medications as a risk factor for adverse drug reactions in the elderly is well established [35,37,38] and polypharmacy as a risk factor for constipation is described in several reviews and studies [6,7,9,41,42,43,44,45]. In contrast, studies by van Dijk et al. [10] and Fosnes et al. [9] of elderly in nursing homes did not find the number of drugs to be a risk factor for constipation. One possible explanation of the difference in findings is that nursing home residents are more likely to have serious comorbidities and are less mobile than community dwelling for the elderly. These conditions are higher relative risk factors for constipation than polypharmacy in the nursing home population.

In this study, the use of one or more drugs with constipation effect was significantly associated with constipation having an odds ratio 2.21 (95% CI 1.20–4.00). The numbers of different drugs with constipation adverse-effect was also associated with constipation having an odds ratio 1.68 (95% CI 1.25–2.26). The results show relative risk appears to be highest with usage of any drug with constipation effect, followed by the number of drugs with constipation effect, and finally the overall number of drugs taken. A past study by van Dijk et al. [10] supports our finding, with usage of drugs exhibiting moderate to strong constipation effects with an odds ratio 1.69 (95% CI 1.33–2.15) [10].

Within specific drug classes, calcium supplements (A12A), ACE inhibitors (C09A) and vitamins (A11) all showed an associative trend with constipation, while opioid (N02A) intake was different between patient and control groups. Opioids and calcium supplements are both widely known to be associated with constipation [46,47]. The role of ACE inhibitors in constipation is more uncertain, diarrhea symptoms are noted as an adverse effect [48] but others report a mixture of constipation and diarrhea symptoms, with more frequent constipation symptoms [49].

Several studies have reported relative risk of constipation from our list of drug groups with constipation adverse-effect: anticholinergic drugs (OR 1.9; 95% CI 1.5–2.5) [50], benzodiazepine derivatives (OR 2.8; 95% CI 1.12–7.04) [9], anticholinergic antidepressants (OR 3.12; 95% CI 1.21–8.03) [51], iron supplements (OR 2.2) and calcium channel blockers (OR 1.9) [52]. The nonsignificant findings of constipation in our study is likely due to low power since relatively few subjects were in each drug group with constipation adverse effects. The majority of studies on polypharmacy focus on nursing home residents, where the average number of medication is higher, 6.0 [9] and 8.9 [10] drugs per person, compared to the community dwelling elderly in this study, 4.7 drugs per person.

We found a few drug classes to be independent protective factors against constipation. Although this protective factor against constipation has not been described before, studies have associated the following drug groups with diarrhea: drugs for peptic ulcer and GERD (A02B) [53], antithrombotic agents (B01A) [54], and antibacterial (J01) [55]; or to relieve symptoms of constipation: analgesics and antipyretics (N02B) [56].

Data for drug usage and constipation in the elderly are scarce; this study is the first to demonstrate their associations within an Australian population. Drugs with constipation effects should be taken into account when pharmacotherapy is needed, with special reference to other known risk factors for constipation: patients’ gender, comorbidity, and mobility. Alternative pharmacotherapy could be considered. For example, the use of newer antidepressants with minor or no anticholinergic activity could be considered as alternatives to antidepressants with anticholinergic side-effects.

The limitations of the present study need consideration. Response rate was lower than desired (34.7%). It was anticipated that this study would have a low response rate due to exclusively focusing only on the elderly population of GWS, composed of culturally and linguistically diverse residents. Questionnaires in multiple languages and reminders to nonrespondents may have improved response rates and the completion of surveys. The low response rate resulted in a small study cohort which may have selective bias and might reduce the external validity of our results. Due to these limitations, we cannot extrapolate these findings to different populations or convincingly prove a causal association between total medication use and constipation. However, the consistency between our results and those published previously is reassuring [23,35,36] and any differences between studies are likely explained by classification factors.

Another limitation of this study is that we did not examine or account for all factors associated with constipation, such as socioeconomic status, education level, ethnicity, weight, diet, physical activity, or predisposing medical conditions. Studies have explored each of these factors in various populations and shown mixed results [22,23,30,57,58,59]. To incorporate all these factors in to a single population study when the significance and role of each is poorly understood is a colossal task. Instead, we chose to explore one key risk factor, medication usage, as it is an area that has been poorly studied in this region and is relevant for health professionals, in the context of the updated Rome III criteria for constipation.

## 5. Conclusions

In summary, constipation is prevalent in the GWS community. Constipation is associated with the number of drugs taken and, in particular, drugs with constipation adverse effects. Therefore, in the elderly with constipation, the focus should be on minimizing prescribing drugs with constipation adverse effect followed by reducing the number of drugs taken.

## Figures and Tables

**Table 1:** Mean (±standard deviation (±SD)) or proportion (±SD) of age, gender and functional constipation for all participants and these values alongside *p*-values for comparisons between participants with and without constipation.

Characteristics	All Participants (*n* = 236)	Mean or Proportion of Participants with Constipation (*n* = 80)	Mean or Proportion of Participants without Constipation (*n* = 156)	*p*-Value
Age (years)	73.8 (6.2)	73.9 (6.6)	73.8 (6.0)	0.79
Gender (Male)	53.8% (0.50)	50% (0.50)	55.7% (0.50)	0.40
Functional constipation (Rome III criteria)	16.9% (0.38)	50% (0.50)	0	-

**Table 2:** Mean (SD) or proportion (±SD) of drug use, number of drugs, regular use of laxatives, use of drugs with constipation adverse effect, number of drugs with constipation adverse effect for all participants and these values alongside *p*-values for comparisons between participants with and without constipation.

	All Participants (*n* = 236)	Participants with Constipation (*n* = 80)	Participants without Constipation (*n* = 156)	*p*-Value
Use drugs	88.9% (0.32)	93.8% (0.24)	85.9% (0.35)	0.07
Number of drugs (mean with ±SD)	4.7 (3.4)	6.2 (3.7)	3.9 (3.0)	<0.001
Laxative regularly	6.8% (0.25)	20.0% (0.40)	0	n.a.
Use drugs with constipation adverse-effect	47.4% (0.49)	59.0% (0.44)	55.7% (0.50)	0.007
Number of drugs with constipation adverse-effect (mean with ±SD)	1.0 (1.0)	1.3 (1.0)	0.8 (0.9)	<0.001
Drugs with constipation adverse effects				
Opioid (N02A; *n* = 7)	3.0% (0.17)	8.8% (0.28)	0	<0.001
Anticholinergic (N04A; *n* = 6)	2.5% (0.16)	5.0% (0.22)	1.3% (0.11)	0.18
Antidepressant (*n* = 16)	6.8% (0.25)	10% (0.30)	5.1% (0.22)	0.18
Calcium Channel Blocker (C08; *n* = 57)	24.2% (0.43)	30% (0.46)	21.2% (0.41)	0.15
Beta Blocking agents (C07; *n* = 41)	17.4% (0.38)	18.8% (0.39)	16.7% (0.37)	0.72
Anti-inflammatory and antirheumatic products, and steroids (M01A; *n* = 16)	6.8% (0.25)	6.3% (0.24)	7.1% (0.26)	1.00
Antineoplastic agents (L01; *n* = 4)	1.7% (0.13)	2.5% (0.16)	1.3% (0.11)	0.61
Iron Preparations (B03A; *n* = 3)	1.3% (0.11)	2.5% (0.16)	0.6% (0.08)	0.27
Antipsychotics (N05A; *n* = 1)	0.4% (0.07)	1.25% (0.11)	0	0.34
Diuretics (C03; *n* = 28)	11.9% (0.32)	17.5% (0.38)	9.0% (0.29)	0.09
Calcium supplements (A12A; *n* = 34)	14.4% (0.35)	15.0% (0.36)	14.1% (0.35)	0.85
Antiarrhythmic (C03; *n* = 3)	1.3% (0.11)	1.3% (0.11)	1.3% (0.11)	1.00
Antihistamine for systemic use (R06; *n* = 10)	4.2% (0.20)	7.5% (0.26)	2.5% (0.16)	0.09
Anxiolytics (N05B; *n* = 1)	0.4% (0.07)	0	0.6% (0.08)	1.00
Antacids (A02A; *n* = 2)	0.8% (0.09)	0	1.3% (0.11)	0.55
Other drugs				
Drugs for peptic ulcer and GERD (A02B; *n* = 63)	26.7% (0.44)	41.3% (0.50)	19.2% (0.40)	0.001
Lipid modifying agents (C10A; *n* = 106)	44.9% (0.51)	51.2% (0.60)	41.7% (0.50)	0.16
Ace inhibitors (C09A; *n* = 37)	15.7% (0.34)	12.5% (0.33)	17.3% (0.38)	0.34
Blood glucose lowering drugs excl. insulins (A10B; *n* = 32)	13.6% (0.34)	13.8% (0.34)	13.5% (0.34)	0.95
Antithrombotic agents (B01A; *n* = 55)	23.3% (0.42)	35.0% (0.48)	17.3% (0.38)	0.003
Angiotensin II antagonists (C09C; *n* = 85)	36.0% (0.49)	43.8% (0.51)	32.1% (0.47)	0.08
Other analgesics and antipyretics (N02B)	12.7% (0.33)	20.0% (0.40)	9.0% (0.29)	0.02
Vitamins (A11; *n* = 58)	24.6% (0.41)	27.5% (0.43)	23.1% (0.42)	0.45
Minerals (A12; *n* = 39)	16.5% (0.36)	21.2% (0.40)	25% (0.42)	0.16

All drugs with known constipation adverse effects are presented, other drugs are only presented if *n* ≥ 25; semi-interquartile range is third quartile–first quartile divided by two; GERD: Gastroesophageal reflux disease.

**Table 3:** Logistic regression models for constipation with number of drugs (Model 1), patients on at least one drug with constipation side effect (Model 2), number of drugs with constipation side effects (Model 3), or groups of drugs (Model 4) as predictors.

Independent Variables	Model 1		Model 2		Model 3		Model 4	
	OR (95% CI)	*p*-Value	OR (95% CI)	*p*-Value	OR (95% CI)	*p*-Value	OR (95% CI)	*p*-Value
Number of drugs	1.24 (1.13–1.35)	<0.001	-	-	-	-	-	-
Drugs with constipation effect (*n* = 112)	-	-	2.21 (1.2–4.0)	0.009	-	-	-	-
Number of drugs with constipation effect	-	-	-	-	1.68 (1.25–2.26)	0.001	-	-
Calcium channel blockers (C08; *n* = 57)	-	-	-	-	-	-	0.62 (0.24–1.58)	0.31
Beta blocking agents (C07; *n* = 41)	-	-	-	-	-	-	1.57 (0.51–4.87)	0.44
Diuretics (C03; *n* = 28)	-	-	-	-	-	-	0.92 (0.26–3.32)	0.904
Calcium supplements (A12A; *n* = 34)	-	-	-	-	-	-	4.67 (0.86–25.29)	0.07
Drugs for peptic ulcer and GORD (A02B; *n* = 63)	-	-	-	-	-	-	0.23 (0.09–0.56)	0.001
Lipid modifying agents (C10A; *n* = 106)	-	-	-	-	-	-	1.00 (0.45–2.22)	1.00
ACE inhibitors (C09A; *n* = 37)	-	-	-	-	-	-	2.88 (0.85–9.76)	0.08
Blood glucose lowering drugs excl. insulins (A10B; *n* = 32)	-	-	-	-	-	-	1.31 (0.40–4.30)	0.66
Antithrombotic agents (B01A; *n* = 54)	-	-	-	-	-	-	0.33 (0.13–0.89)	0.03
Angiotensin II antagonists (C09C; *n* = 85)	-	-	-	-	-	-	1.09 (0.44–2.66)	0.858
Other analgesics and antipyretics (N02B; *n* = 30)	-	-	-	-	-	-	0.35 (0.10–1.95)	0.05
Vitamins (A11; *n* = 58)	-	-	-	-	-	-	2.92 (0.92–9.27)	0.07
Mineral supplements (A12; *n* = 39)	-	-	-	-	-	-	0.41 (0.13–1.30)	0.13

Note: Age and gender have been included in all analysis regardless of significance; only drug groups with *n* ≥ 25 are considered. ACE: Define; CI: Confidence interval; OR: Odds ratio.
